# Safety Culture in the Maternity Units: a census survey using the Safety Attitudes Questionnaire

**DOI:** 10.1186/1472-6963-11-238

**Published:** 2011-09-27

**Authors:** Vasilios Raftopoulos, Nicos Savva, Maria Papadopoulou

**Affiliations:** 1 Cyprus University of Technology, Nursing Department, Mediterranean Research Centre for Public Health and Quality of Care, Nicosia, Cyprus; 2 Cyprus University of Technology, Nursing Department, Nicosia, Cyprus

**Keywords:** patient safety, maternity care, quality care, safety culture, safety attitudes

## Abstract

**Background:**

Patient safety has been a priority for many societies and health care systems in the last decades. Identification of preventable risks and aversion of potentially unsafe situations and fatal complications in maternity units is life saving. The explicit need to focus on quality of care underpins the aim of the study to initially evaluate the safety culture and teamwork climate in the public Maternity Units of the 5 Regional Hospitals in Cyprus as measured by a validated safety attitudes tool.

**Methods:**

Data were collected from 140 midwives working in the public sector all over Cyprus by the Greek Version of the Safety Attitudes Questionnaire-Labor version.

**Results:**

One hundred and six (75.71%) registered midwives completed the questionnaire fully. The median of total work experience as a registered midwife was 3 years (IQR: 2-18.25); whereas the median of total working experience in the nursing and maternity units was 5 years (IQR: 2-21.75). Experienced midwives rated the following domains higher: team work, safety climate, job satisfaction and working conditions as opposed to the midwives with less experience. Additionally those with a longer working life in the current maternity units rated these domains higher: safety climate, job satisfaction and working conditions as opposed to the less experienced midwives.

**Conclusions:**

The high mean total score on team work and safety climate in the more experienced group of midwives is a predominant finding for the maternity units of Cyprus. In Cyprus where facilities are small in size and midwives know each other, share more responsibility towards patient safety. It could be suggested that younger midwives need more support and teamwork practice to enhance the safety and teamwork climate towards self-confidence.

## Background

The Cyprus public Maternity services consist of five maternity units that refer to approximately 140 beds, delivery rooms and prenatal clinics. The number of registered practicing midwives in the public sector is 171 [1]. According to the 2007 annual report of the Ministry of Health of Cyprus [2], midwives fulfill the International Confederation of Midwives "Definition of the Midwife" [3] and the requirements of the European Directive 2005/36/EC that regulates the midwifery profession [4]. The birth rate in Cyprus is 11.6 [5] with high medicalization of childbirth as only 50.6% of the women in Cyprus give birth without an obstetric intervention [6].

Patient safety has been placed high on the societal agenda in the last decade although *Premium non nocere *('first do no harm') has been a maxim of healthcare professionals for many centuries. The World Health Organization [7], has defined patient "unsafety" as "*a process or act of omission or commission that resulted in hazardous healthcare conditions and/or unintended harm to the patient"*.

Timely emergency obstetric services, adequate communication and skilled personnel working as a team in a good and safe environment are recommended in order to prevent the 15% of pregnant women in all population groups who experience potentially fatal complications [7]. Maternal morbidity and mortality which can also be prevented in functioning health care systems are defined as the best indicators of overall health system performance evaluation; therefore, sustaining and scaling-up essential health interventions and addressing critical gaps in maternal care and maternity staff practices that are essential to improve women's health [8-10].

Evidence from WHO and the recent Healthcare Commission investigation reports [11-16]; suggested that a poor working relationship between healthcare professionals, including lack of support and possible bullying, can lead to dysfunctional teams which can further compromise women's and neonates health. Effective systems of communication between the multidisciplinary team members and each discipline have been major recommendations of the "Safer Childbirth" program [17,18].

The WHO Regional Office for Europe identified that prevention, identification, and management of risk in obstetrics demand multidisciplinary and multiprofessional team training such as "Acute Life-Threatening Events Recognition and Treatment", transparent clinical governance, multidisciplinary team work, communication as keystone of good clinical practice, adequate staffing levels, standardized practices, and auditing systems [19]. In environments with established interprofessional team training and support from senior clinical and management leaders, teambuilding and safety attitudes are positive [20]. The overall safety climate reduces the rate of adverse obstetric events, so the assessment and improvement of the safety climate is the first step to reduce patient risk (injury, liability losses) and create a culture of safety [21]. Attitude represents the degree of an individual's like and dislike of an item [22]; while a good safety climate is characterized by a collective commitment of care and concern, whereby employees share similar positive perceptions about organizational safety features [23]. To conduct this initial assessment, the SAQ-Labor version which has been used in several studies [20,21,24,25] was considered as a valid measurement tool. Whereas safety attitudes are necessary, they are not sufficient enough to improve clinical performance and perinatal safety [26] as leadership and teamwork are essential ingredients [27,28].

The growing overall aversion of potentially unsafe situations and the explicit focus of healthcare professionals on the quality of care, underpins the need of this study; to evaluate the safety attitudes and teamwork climate in the care of birthing mothers and neonates in the public maternity units of Cyprus. Additionally, more than eight out of ten citizens in Cyprus feel it is likely they will be harmed by hospital care [9].

The European Network for Patient Safety (EUNetPaS) that was officially inaugurated on February 28, 2008 in Utrecht sought to establish a covering network for all 27 EU member states to encourage and strengthen cooperation in the field of patient safety. According to the latest report by EUNetPas, Cyprus is among the countries that still have not adopted a tool for measuring patient safety, making this survey a pioneer in this field [29]. In the Eurobarometer survey, the issue of patient safety is quite visible in figures, as 50% of the respondents in the 27 EU member states replied that they "feel they would suffer from an adverse event" if hospitalized. However, only 9% believe that it is very likely to happen. Of particular interest are the figures for Cyprus, where 81% feel that risk. Greece was ranked first by 83% and Latvia by 75%, while Austria and Germany are among the countries where citizens feel that this is unlikely to happen with 19% and 31% respectively [9].

Taking the above into consideration and keeping in mind that safety culture and teamwork climate have not been examined within the population of midwives in Cyprus, a research study was undertaken to assess the safety climate as measured by a validated safety attitude tool.

### Aim

The aim of the study was to explore the factors that affect the safety attitude and teamwork climate of Cyprus maternity units and Cypriot midwives.

## Method

Subjects who consented to participate were registered midwives, working in the public maternity units. Potential participants were recruited on the basis of their availability. They were approached by the researchers and were given a detailed explanation of the purpose and aim of the study. An informed consent was obtained from those who agreed to participate and they were asked to complete the questionnaire. One hundred and seventy one midwives work in the public sector in Cyprus [1]. Thus by using a random stratified sampling method and taking into account geographical allocation, specialty and type of employment, 140 midwives working in the public sector were approached. One hundred and fifteen questionnaires were completed and returned, of which seven were deemed incomplete and therefore were not included in the study. The final sample consisted of 106 midwives.

### Measurement tool

The questionnaire was divided into two major parts. The first contained: demographic information and questions related to the level of occupational stress and fatigue. To assess perceived job exhaustion we have used the general polar question: "Do you feel generally exhausted some times?" yes/no. To assess perceived occupational stress we used the general polar question: "Do you feel that your profession is a source of stress to you?" Midwives were also asked to rate perceived global quality of maternity care and global job satisfaction, by using a continuous scale that ranged from 0-10. The participants were also asked to answer to the question: "how safe do you consider patients feel in your maternity unit?" using the same rating scale 0-10. The second part of the questionnaire introduced participants to the Safety Attitudes Questionnaire (SAQ) Labour version format developed by Sexton [24]. The SAQ consists of items both from the Flight Management Attitudes Questionnaire and new items generated on the basis of Vincent's framework for analysing risk and safety [30] and Donabedian's conceptual model for assessing quality in health care [31].

The Safety Attitudes Questionnaire (SAQ) Labor version format elicits health care workers' attitudes through 6 domains: teamwork climate, job satisfaction, perceptions of management, safety climate, working conditions, and stress recognition. The response to each of the items is a 5-point Likert scale (1 = *disagree strongly*, 2 = *disagree slightly*, 3 = *neutral*, 4 = *agree slightly*, 5 = *agree strongly*). To calculate the 100 pt scale score (e.g., teamwork climate) for an individual respondent we have followed the equation proposed by Sexton. Thus the total score of the subscales could range from 0 to 100. The scores obtained represented individual perceptions with higher scores reflecting more favourable perceptions of the item. The corresponding author obtained a permission to use the questionnaire in the Greek language.

The translation of the SAQ-Labor version into Greek included four steps: forward and back translation, and expert panel review. Two researchers translated the English version of the SAQ-Labor into Greek (table 1). Word-for-word translation was avoided because this form of translation does not account for linguistic and cultural differences [32]. These two translations were reviewed by a panel of experts to determine the final version, and this was subsequently back translated into English. Expert validity is a form of content validity, which is demonstrated by asking experts to review the content of the instrument. The minimum number of experts required is five [33]. Our panel consisted of three midwives and two academics with an expertise in health care quality management who reviewed the questionnaire for clarity and offered relevant feedback. The panel of experts reviewed the back-translated version of the SAQ-Labor version, and their comments were evaluated to ensure exact replication of the original instrument. The aims were to ensure cross-cultural comparability and to adapt the scale for the Cypriot population. Minor corrections were agreed by the reviewers, concerning the periphrastic yield of certain words from English into Greek, without essential alteration of the meaning of sentences or the meaning of the instrument as a whole.

**Table 1:** Greek translation of the SAQ-Labor Questionnaire

Ερωτήσεις	Διαφωνώαπóλυτα	Διαφωνώκπως	Ουδέτερος/η	Συμφωνώκάπως	Συμφωνώαπόλυτα
**35. Είναι εύκολο για το προσωπικό σε αυτό το Μαιευτικό Τμήμα να κάνει ερωτήσεις όταν δεν κατανοεί κάτι**	**□**	**□**	**□**	**□**	**□**

**34. Έχω την υποστήριξη που χρειάζομαι από το υπόλοιπο προσωπικό για να φροντίζω τους ασθενείς**	**□**	**□**	**□**	**□**	**□**

**3. Οι εισηγήσεις των μαιών λαμβάνονται υπόψη σε αυτό το Μαιευτικό Τμήμα**	**□**	**□**	**□**	**□**	**□**

**24. Σε αυτό το Μαιευτικό Τμήμα είναι δύσκολο να μιλήσω εάν αντιληφθώκάποιο πρόβλημα που αφορά στη φροντίδα των ασθενών**	**□**	**□**	**□**	**□**	**□**

**30. Οι διαφωνίες σε αυτό το Μαιευτικό Τμήμα επιλύονται κατάλληλα (π.χ. όχι ποιος έχει δίκαιο αλλά τι είναι προς το συμφέρον του ασθενή)**	**□**	**□**	**□**	**□**	**□**

**38. Οι ιατροί και οι μαίες σε αυτό το Μαιευτικό Τμήμα εργάζονται μαζί σαν μια καλά συντονισμένη ομάδα**	**□**	**□**	**□**	**□**	**□**

**21. Η εργασιακή κουλτούρα σε αυτό το Μαιευτικό Τμήμα διευκολύνει τη μάθηση από τα λάθη των άλλων**	**□**	**□**	**□**	**□**	**□**

**5. Τα λάθη αντιμετωπίζονται κατάλληλα σε αυτό τον κλινικό χώρο**	**□**	**□**	**□**	**□**	**□**

**28. Γνωρίζω τις κατάλληλες διαδικασίες για να υποβάλω ερωτήματα σχετικά με την ασφάλεια των ασθενών στο Μαιευτικό Τμήμα**	**□**	**□**	**□**	**□**	**□**

**20. Ενθαρρύνομαι από τους συναδέλφους μου να αναφέρω οποιεσδήποτε ανησυχίες μπορεί να έχω σχετικά με την ασφάλεια των ασθενών**	**□**	**□**	**□**	**□**	**□**

**11. Λαμβάνω την κατάλληλη ανατροφοδότηση για την απόδοση μου στην εργασία**	**□**	**□**	**□**	**□**	**□**

**4. Θα ένιωθα ασφαλής αν νοσηλευόμουν σε αυτόν τον κλινικό χώρο**	**□**	**□**	**□**	**□**	**□**

**12. Σε αυτό τον κλινικό χώρο είναι δύσκολο να συζητάς τα λάθη**	**□**	**□**	**□**	**□**	**□**

**15. Αυτό το Νοσοκομείο είναι ένα καλό μέρος για να εργαστεί κάποιος**	**□**	**□**	**□**	**□**	**□**

**29. Είμαι περήφανος/η που εργάζομαι σε αυτό το Νοσοκομείο**	**□**	**□**	**□**	**□**	**□**

**8. Όταν εργάζεσαι σε αυτό το νοσοκομείο είναι σαν αν είσαι μέλος σε μια μεγάλη οικογένεια**	**□**	**□**	**□**	**□**	**□**

**41. Το ηθικό σε αυτό το Μαιευτικό Τμήμα είναι υψηλό**	**□**	**□**	**□**	**□**	**□**

**2. Μου αρέσει η δουλειά μου**	**□**	**□**	**□**	**□**	**□**

**25. Όταν ο φόρτος εργασίας μου αυξάνεται επηρεάζεται αρνητικά η απόδοση μου**	**□**	**□**	**□**	**□**	**□**

**32. Είναι πιθανότερο να κάνω λάθη όταν επικρατούν τεταμένες ή εχθρικές συνθήκες στην εργασία μου**	**□**	**□**	**□**	**□**	**□**

**16. Η κούραση επηρεάζει αρνητικά την απόδοσή μου κατά τη διάρκεια επειγόντων περιστατικών (π.χ. καρδιοαναπνευστική αναζωογόνηση, αιμορραγία)**	**□**	**□**	**□**	**□**	**□**

**31. Είμαι λιγότερο αποτελεσματικός/ή στη δουλειά όταν είμαι κουρασμένος/η**	**□**	**□**	**□**	**□**	**□**

**17. Η Διοίκηση του Νοσοκομείου δεν διακινδυνεύει (εν γνώσει της) την ασφάλεια των ασθενών**	**□**	**□**	**□**	**□**	**□**

**10. Η Διοίκηση του Νοσοκομείου στηρίζει τις καθημερινές μου προσπάθειες**	**□**	**□**	**□**	**□**	**□**

**26. Ενημερώνομαι επαρκώς και έγκαιρα για διάφορα πράγματα που συμβαίνουν στο Νοσοκομείο και μπορούν να επηρεάσουν την εργασία μου**	**□**	**□**	**□**	**□**	**□**

**18. Η στελέχωση είναι επαρκής σε αυτό το Μαιευτικό Τμήμα που εργάζομαι σε σχέση με τον αριθμό των ασθενών**	**□**	**□**	**□**	**□**	**□**

**7. Όλες οι απαραίτητες πληροφορίες για τις διαγνωστικές και τις θεραπευτικές αποφάσεις είναι διαθέσιμες σε εμένα σε καθημερινή βάση**	□	□	□	□	□

**22. Αυτό το Νοσοκομείο αντιμετωπίζει με εποικοδομητικό τρόπο το προβληματικό προσωπικό**	□	□	□	□	□

**42. Οι εκπαιδευόμενοι υπό την ευθύνη μου επιτηρούνται επαρκώς**	□	□	□	□	□

**6. Αυτό το Νοσοκομείο κάνει καλή δουλειά στην κατάρτιση του νέου προσωπικού**	□	□	□	□	□

Once the questionnaire was translated and adapted for the Cypriot population, it was tested by a purposive sample of ten midwives who were lay persons. The participants wrote comments for each question regarding the clarity or ambiguity of the meaning, including whether the questions were culturally acceptable, whether the wording was appropriate, and how easy/difficult it was to understand the language used. Minor corrections were made to the questionnaire following their suggestions.

### Ethical issues

The protocol of the study was reviewed by the Cyprus National Bioethics Committee and approved by the Cyprus Ministry of Health. Health care professionals were free to participate in or withdraw from the study at any time, anonymity of data was preserved, and the data that emerged was kept safely. Completion of a questionnaire was considered as an informed consent for participation. A cover letter with information on the aim of the study accompanied the questionnaires.

### Data analysis

All the items were coded and scored, and the completed questionnaires were included in the data analysis set. PASW-18 was used to analyze the data. The chi-square test was used to explore the existence of a statistically significant relationship between the categorical variables. The t-test was used to assess whether the means of two groups were statistically different from each other. Values < 0.05 were considered to be statistically significant, unless otherwise stated. Internal consistency of the SAQ-Labor scale was assessed by calculating Cronbach's alpha. A series of linear regression analyses were undertaken for each of the 6 average domain scores using unit choice, age group, perceived quality of maternity care, profession selection, job exhaustion, level of occupational stress, the dichotomous grading of the current hospital in comparison with other hospitals, and perceived patients' safety at the Maternity Ward as independent variables, with backward stepwise deletion of nonsignificant variables. Data collection was conducted between October and November of 2010.

## Results

One hundred and six (75.71%) registered midwives completed the questionnaire. The mean age of the participants was 38.65 ± 10.09 (range 21-58 years old). The median of total work experience as a registered midwife was 3 years (IQR: 2-18.25); whereas the median of total working experience in the nursing and maternity units was 5 years (IQR: 2-21.75). The sample was homogenous in terms of gender (i.e 100% female responders) as there are no male midwives practicing in public hospitals in Cyprus. The majority of the participants were married (n = 79, 77.5%) while 19 (18.6%) were single, 3 (2.9%) were partners and one (1%) was divorced. In order to assess perceived global quality of care provided in the labour unit, an ordinal scale ranging from 0-10 was introduced, the mean score was 7.57 ± 2.24. The age of the participants correlated strongly (r = 0.835; p < 0.001) with the perceived global quality of care provided in the maternity unit. As for the perception of quality of services that midwives personally provide in association with their age there was also a strong correlation (r = 0.45; p < 0.001). In addition, age and job satisfaction in the maternity units, correlated strongly (r = 0.49; p < 0.001). For the vast majority (80.4%) of the participants working in a particular maternity unit was their own choice. T test analysis did not reveal any statistical difference between choosing the unit and the other variables used in the first part of the questionnaire.

As seen in Table 2 experienced midwives rated the following domains: team work, safety climate, job satisfaction and working conditions higher as opposed to the midwives with less work experience. Additionally those with a longer working life in the current maternity units scored the factors: safety climate, job satisfaction and working conditions higher as opposed to the less experienced midwives.

**Table 2:** mean scores in the SAQ-Labor subscales

SAQ subscale		Total work experience as a Midwife			Total work experience in this Department		
	**Mean (SD)**	**Less experience**	**More experience**	**p**	**Less experience**	**More experience**	**p**

Team Work	57.95 (21.17)	55.37 (18.58)	64.24 (20.09)	0.025	54.76(19.22)	65.07 (19.93)	0.12
Safety Climate	55.82 (18.97)	51.51(15.22)	64.29 (17.24)	< 0.001	52.19 (14.39)	64.74 (17.58)	< 0.001
Job Satisfaction	66.20 (22.64)	63.08 (20.34)	75.42 (18.01)	0.002	63.27 (19.96)	74.57 (19.96)	0.007
Perception of Management	52.14 (19.69)	52.64 (16.98)	53.52 (20.98)	0.821	51.91 (17.17)	55.72 (20.96)	0.334
Working Conditions	55.03 (20.83)	53.00 (16.34)	63.15 (18.47)	0.005	53.57 (14.14)	64.76 (18.12)	0.01
Stress Recognition	50.64 (18.77)	51.44 (16.30)	52.47 (18.03)	0.765	52.68 (17.35)	51.86 (17.48)	0.819

Using the median (35 years old) of the participant's age t-test was performed in order to investigate any relation between their age and their mean total scores in the safety attitudes questionnaire's sub scales. As presented in Table 3 maternity unit choice is irrelevant to any of the six SAQ's domains. A statistically significant difference (p = 0.02), was observed in terms of the safety climate perception with those aged above the median age (35-58 years old) scoring higher. People in this group perceived work climate as safer whereas the people below the median age felt that the safety climate in the maternity services is not so strong. Power analysis performed retrospectively indicated that comparing groups on the basis of median levels of the variables under investigation (i.e. a sample size of about 53 per group) the study has at least 80% power to detect a 10 unit difference in the total scores at the 5% significance.

**Table 3:** Mean SAQ-Labor scores by social and demographic characteristics

Variable	Team Work	Safety Climate	Job satisfaction	Perception of Management	Working Conditions	Stress Recognition
**Unit Choice**						
Yes (my own choice)	50.09 ± 20.82	57.31 ± 19.69	66.90 ± 23.09	51.80 ± 20.44	56.25 ± 21.41	61.06 ± 23.00
No	52.58 ± 18.56	55.78 ± 15.25	63.33 ± 20.93	53.57 ± 16.60	56.55 ± 16.70	56.25 ± 17.11
	p = 0.242	p = 0.701	p = 0.498	p = 0.677	p = 0.945	p = 0.289
**Age group**						
< 35 years old	55.65 ± 17.72	50.63 ± 15.29	61.56 ± 21.01	50.83 ± 16.07	50.28 ± 18.22	58.28 ± 18.31
> 35 years old	62.50 ± 20.93	61.47 ± 17.95	71.44 ± 20.65	55.17 ± 20.13	60.34 ± 20.10	53.37 ± 17.31
	p = 0.84	p = 0.02	p = 0.22	p = 0.40	p = 0.24	p = 0.11
**Perceived quality**						
< median (0-8)	57.96 ± 19.706	52.43 ± 18.52	64.23 ± 22.43	50.32 ± 18.70	53.04 ± 20.34	49.36 ± 18.75
> median (9-10)	57.92 ± 24.96	64.64 ± 18.50	71.33 ± 22.74	56.88 ± 21.67	60.21 ± 21.55	53.96 ± 18.74
	p = 0.99	p = 0.003	p = 0.151	p = 0.151	p = 0.122	p = 0.259
**Profession selection**						
No	56.94 ± 21.26	55.27 ± 16.07	63.33 ± 20.93	53.57 ± 16.60	52.98 ± 18.60	52.08 ± 19.39
Own decision	58.19 ± 21.26	55.95 ± 19.69	66.90 ± 23.09	51.80 ± 20.44	55.53 ± 21.40	50.29 ± 18.72
	p = 0.811	p = 0.869	p = 0.498	p = 0.677	p = 0.587	p = 0.704
**Job exhaustion**						
No	60.28 ± 27.85	58.10 ± 27.24	66.00 ± 31.40	56.67 ± 29.26	57.08 ± 32.55	54.58 ± 27.49
Yes	57.38 ± 20.08	55.32 ± 17.50	66.30 ± 21.22	51.36 ± 17.89	54.48 ± 18.49	49.73 ± 16.98
	p = 0.704	p = 0.707	p = 0.972	p = 0.505	p = 0.767	p = 0.517
**Job induced stress**						
No	56.52 ± 22.82	55.15 ± 22.21	66.18 ± 27.49	51.47 ± 23.48	56.62 ± 22.87	54.23 ± 19.74
Yes	57.75 ± 20.0 9	55.41 ± 16.91	65.83 ± 20.10	51.91 ± 17.60	53.47 ± 19.50	48.78 ± 18.15
	p = 0.805	p = 0.952	p = 0.948	p = 0.923	p = 0.492	p = 0.179
**Hospital Grading ordinal scale in comparison with other hospitals work**						
< median (0-7)	55.03 ± 21.878	52.28 ± 19.88	62.24 ± 23.07	51.94 ± 19.52	51.40 ± 22.55	49.57 ± 19.86
> median (8-10)	61.33 ± 20.01	59.93 ± 17.01	70.80 ± 21.43	52.38 ± 20.08	59.25 ± 17.95	51.88 ± 17.55
	p = 0.121	p = 0.034	p = 0.48	p = 0.910	p = 0.047	p = 0.523
**Patients safety at your Maternity Ward**						
< median (0-8)	54.75 ± 20.66	52.29 ± 18.92	59.92 ± 21.72	49.71 ± 19.62	49.90 ± 20.93	49.61 ± 18.53
> median (9-10)	62.59 ± 21.27	60.96 ± 18.05	75.34 ± 20.97	55.68 ± 19.47	62.50 ± 18.48	52.13 ± 19.24
	p = 0.060	p = 0.018	p < 0.001	p = 0.122	p < 0.001	p = 0.499

T-test analysis

Also people who stressed that global quality in the maternity services is high, scored SAQ's perceived safety climate (p = 0.003) higher. Profession selection was not associated with the mean scores of the SAQ domains. It is worth mentioning that all subjects whether they stated that they suffer from job exhaustion or not, they did not think that job exhaustion can affect team work, safety climate, job satisfaction from their job, or the working conditions in the maternity unit. The results are similar for the question: "do you believe your job is a source of anxiety".

Those who replied that they have previously worked at a different hospital and gave a high grading score for their current setting had a statistically significant (p = 0.034) better perception of the safety climate and they appeared more satisfied with their job even thought the difference was not statistically significant (p = 0.48) and they believed that their current working conditions are better (p = 0.047). Furthermore, for those who gave the highest grade (i.e. above median) for the perception of safety in their maternity unit, safety climate, job satisfaction and working conditions are perceived as much better for this group.

The items with highest scores were the following: "I like my job" (82.18), "I would feel safe being treated here as a patient" (69.91), "it is easy for personnel in this clinical area to ask questions when there is something they do not understand" (67.36), "Morale in this clinical area/unit is high" (66.90), "this hospital is a good place to work" (65.97). The items with the lower scores were: "this hospital deals constructively with problem physicians and personnel" (42.59), "hospital administration supports my daily efforts" (44.68), "I receive appropriate feedback about my performance" (46.06), "the levels of staffing in this clinical area are sufficient to handle the number of patients" (47.69), "I am more likely to make errors in tense or hostile situations" (47.69).

### Regression analyses

Linear regression analysis using the variables in Table 3 as independent variables, revealed that the predictors of: (1) safety climate were the age group (beta = 9.54; p = 0.008) perceived quality of maternity care (beta = 8.60; p = 0.034) and the level of occupational stress (beta = 6.14; p = 0.034). All the independent variables accounted for 23.2% of the variation in safety climate, (2) teamwork was job exhaustion (beta = -12.85; p = 0.046). All the independent variables accounted for the 11.7% of the variation in teamwork, (3) job satisfaction was midwives' answer to the question how safe do you consider patients feel in your maternity unit (beta = -12.85; p = 0.046). All the independent variables accounted for 17.1% of the variation in job satisfaction, (4) stress recognition: none of the variables was a predictor although the independent variables accounted for 4.2% of the variation in stress recognition, (5) perception of management was job exhaustion (beta = -13.08; p = 0.034). All the independent variables accounted for 8.2% of the variation in perception of management, and (6) working conditions were age group (beta = 8.23; p = 0.049), and midwives' answer to the question how safe do you consider patients feel in your maternity unit (beta = 9.57; p = 0.031). All the independent variables accounted for 15.4% of the total variance in working conditions.

### Reliability analysis

Internal consistency of the SAQ-Labor 57-item version proved very good as Cronbach's alpha was 0.93 and 0.86 for the 30-item version.

## Discussion

This research has explored the factors that affect the safety attitudes of Cypriot midwives. The strength of the study was its representativeness, since 77% of all the midwives who work in the public sector in Cyprus were surveyed. To our knowledge, this is the first published nationwide research in the field in Cyprus.

In the current study the mean total score of Teamwork subscale was 57.95 and for Safety Climate was 55.82. In the relevant literature for the maternity services mean scores ranged from 55.4 to 76.1 for the Teamwork Climate and from 55.4 to 74 for the Safety Climate [20,21,24]. Results are in agreement but since we had no intervention such as multi-professional team training [25] the interpretation of this initial assessment of safety and teamwork climate can be attributed to the samples' specific characteristics. All the safety climate domains in our research with the exception of job satisfaction domain for the experienced midwives had a 100 point score below 75 that is considered to be a positive score. These results are not indicative of a positive safety climate.

The low Safety Climate scores reflect the perceptions of midwives considering safety in their units and the way patient safety issues and adverse events are reported and are managed. The vast majority of the midwives answered that they would feel safe being treated in their unit as a client. Their answer suggests that there is a hidden parameter for perceived low Safety Climate rather than an actual compromise. The negative safety climate domain could be possibly attributed to the low commitment of the units' leadership toward patient safety [24], and on how safety issues are managed. Therefore, infrastructure and leadership attitudes towards handling of medical errors and learning from adverse events are important aspects that must be addressed to improve safety climate in the maternity units in Cyprus. Lack of feedback and reduced motivation amongst the midwives to report errors or adverse events influences the prevailing Safety Climate. Furthermore many maternity units lack of clinical guidelines, standard communication practices, knowledge of chain of command, and quality assurance mechanisms that may interfere to midwives' safety climate attitudes [21] although Cypriot midwives acknowledged that there is a linkage between perceived quality of maternity care and safety climate.

In this research the mean total scores for job satisfaction, stress recognition, perceptions of management and working conditions were subsequently, 66.20, 50.64, 52.14, and 55.03. Comparing our scores with those in another survey in the maternity services our sample rated higher stress recognition and perceptions of management subscales, as opposed to job satisfaction that was low and working conditions that were very low [20]. Low scores in working conditions were also identified by others [20], who also identified a need for enhancement of communication tools as integral components for reduction of obstetric adverse events. Working conditions domain relate to several factors such as training and supervision. The more experienced midwives rated this domain statistically significant higher. Poor teamwork or communication in the maternity care team and the lack of adequate supervision of junior midwives may explain this finding that must be addressed to improve the safety climate in these settings. Less experienced midwives may fail to recognise or escalate care when safety problems arise and they need an ongoing supervision and training for continuous professional career development.

In our sample teamwork climate was actually low in the less experienced midwives. A possible explanation could be that the newly qualified staff because they are novice in experience and teambuilding qualities, they lack several characteristics of the professionals with well established competencies of the profession, especially in emergencies. It takes considerable time and experience is needed to develop the level and quality of collaboration and communication between health care providers in order to function as a cohesive team in a clinical environment. A team needs familiarization of its team members, experience and other common factors such as trust, professional beliefs, role and job in an organization and perception of collaboration [24]. In many cases the level of collaboration amongst medical doctors and midwives is questionable. Variations in the perception of the quality of collaboration and communication between health care professionals working in the same unit as in our case have been also reported previously and are considered to be a barrier to cohesive teamwork [24]. Therefore, improvement in teamwork climate and health outcomes enhanced with team training [20,21] must be considered. In the future multi-professional team training and team building practices in Cypriot public facilities must ensure improvement of safety and teamwork climate as well as efficiency and better health outcomes. Midwifery management should take into consideration, of implementing team building events and workshops towards improving interpersonal relationships and team work. Especially less experienced midwifes need support and appropriate feedback in order to engage and start working more constructively as part of a valued team. The role of senior leadership as well as the one of the maternity unit safety midwife is a key element to designing, fostering, and nurturing a culture of safety [25]. Engaged senior leaders are critical to an organization's successful development of a culture of safety. Engaged leaders drive the culture by designing strategy and building structure that guides safety processes and outcomes identified administrative leadership as one of the most significant facilitators for establishing and promoting a culture of safety.

The items that received the highest mean scores were: "I like my job", "I would feel safe being treated here as a patient", "it is easy for personnel in this clinical area to ask questions when there is something they do not understand", these results resemble other studies findings [20]. Despite the cultural differences that may exist between the two samples, the similarity of the findings is indicative of the midwives' views and attitudes on safety. For the 51.7% of the participants the levels of staffing in this clinical area are not sufficient to handle the number of patients. Our percentage is low compared to Siassakos (85.7%) who identified that high workload and insufficient staffing did not correlate negatively with job satisfaction [20]. In the current research positive job satisfaction scores have been reported mainly from the more experienced midwives. Besides, there was a linkage between midwives' job satisfaction and the way they considered that their patients feel safe being treated in their unit. It could be stressed that Cyprus midwives' job satisfaction relates to patient safety. It can be inferred that working in small units where everybody know each other overcomes poor staffing and working conditions effects; which furthermore, increase the tolerance of inadequate working conditions and safe staffing levels that might underpin a risk factor for the pregnant as opposed to the large hospitals [22]. Correlation of age with perceived global quality of maternity services and job satisfaction could also be attributed to the sample and facilities characteristics.

In our research perception of management scores were low both in the experienced and in the less experienced midwives. This domain includes factors relating to the management of the midwives, the leadership and the equipment. Midwives' job exhaustion is postulated to be linked with low perception of management scores. It seems that poor staffing combined with high workload and extended working hours in Cyprus maternity units' increases self reported job exhaustion of midwives that leads to low perception of management. Management decisions that are related to adequate staffing and the availability of the necessary equipment are important to ensure a safety climate [24].

## Conclusions

The safety climate in the maternity settings was negative across all six safety climate domains examined. The higher mean total score on team work and safety climate in the more experienced group of midwives is a predominant finding for the maternity units of Cyprus. It could be suggested that younger midwives need more support and teamwork practice, in a friendly environment, to enhance the safety and teamwork climate through experience and self-confidence.

Effective teamwork is critical in high-risk settings and maternity units especially Labour and Delivery where transition to an emergency is rather quickly [30]. Commitment to a patient safety culture through daily practice of teamwork, communication, collaboration and strong leadership for providers is needed. Lower scores in administrative support, feedback, staffing levels and communication, can be improved by team training on communication among caregivers, between units and management. Our results emphasize the need for a national plan on quality assurance of the provided maternity services in Cyprus. Our attempt represents an initial assessment of the safety and teamwork climate in the public maternity units. Further work is needed to improve this climate, to establish benchmarks and best practices in order to improve health outcomes of mothers and neonates.

### Limitations

A limitation of the study was that the survey was conducted among the midwives who work in the public sector. Sample could include private sector as a nationwide initial assessment of the maternity services. Assessment of the private sector would probably have yielded substantially different results. There was also a lack of qualitative data. In Cyprus the Maternity Units are unified and they include all the sections of care: outpatients, antenatal, intranatal, postnatal and neonatal care, using a rotation schedule. The same midwifery personnel works throughout the whole sections of care. Thus the midwives perceive a global view of quality of care provided. Perhaps in other countries, with larger maternity units the results could be confounded by the setting or other factors. Another limitation is that the results of the current study reflect the views and the attitudes of the respondents who chose to participate in the research and firm conclusions from the measurement of safety climate cannot be drawn from these findings alone.

## Competing interests

The authors declare that they have no competing interests.

## Authors' contributions

VR conceived, designed, acquired the data, coordinated the study, wrote and revised the manuscript, analysed the data and interpreted the results, NS contributed to the writing of the results and participated to the data analysis, MP contributed to the writing of the background and the discussion. All authors read and approved the final manuscript.

## Pre-publication history

The pre-publication history for this paper can be accessed here:

http://www.biomedcentral.com/1472-6963/11/238/prepub
